# Yield and Production Gaps in Rainfed Wheat, Barley, and Canola in Alberta

**DOI:** 10.3389/fpls.2015.00990

**Published:** 2015-11-17

**Authors:** Tejendra Chapagain, Allen Good

**Affiliations:** ^1^Department of Plant Agriculture, University of Guelph, GuelphON, Canada; ^2^Department of Biological Sciences, University of Alberta, EdmontonAB, Canada

**Keywords:** yield gaps, actual yield, attainable yield, maximum attainable yield, management gap, genetic gap, rainfed

## Abstract

Improving crop yields are essential to meet the increasing pressure of global food demands. The loss of high quality land, the slowing in annual yield increases of major cereals, increasing fertilizer use, and the effect of this on the environment all indicate that we need to develop new strategies to increase grain yields with less impact on the environment. One strategy that could help address this concern is by narrowing the yield gaps of major crops using improved genetics and management. The objective of this study was to determine wheat (*Triticum* spp. L.), barley (*Hordeum vulgare* L.), and canola (*Brassica napus* L.) yields and production gaps in Alberta. We used 10 years of data (2005–2014) to understand yield variability and input efficiency at a farmers’ specified level of management, and the yield potential under optimal management to suggest appropriate pathways for closing yield gaps. Significant management gaps were observed between attainable and actual yields of rainfed wheat (24%), barley (25%), and canola (30%). In addition, genetic gaps (i.e., gaps due to genetic selection) in wheat, barley, and canola were 18, 12, and 5%, respectively. Genetic selection with optimal crop management could increase yields of wheat, barley, and canola significantly, with estimated yield gains of 3.42, 1.92, and 1.65 million tons, respectively, each year under rainfed conditions in Alberta. This paper identifies yield gaps and offers suggestions to improve efficiency in crop production.

## Introduction

Improving crop yields is essential to meet the increasing demand for food driven by the increasing population and income growth in the 21st century. One strategy that could address this concern is by quantifying the production capacity of farmland to identify ways to increase the yield of major crops (Patrignani et al., 2014). This can be achieved by using high yielding management practices (Yang et al., 2008), and closing yield gaps between farmers’ actual yield and potential yield (Cassman et al., 2003; Licker et al., 2010; Tilman et al., 2011; Mueller et al., 2012). Miminizing yield gaps in major crops by using optimal management practices may lead to improvements in production, while offering both environmental benefits and economic value. Assessing the yield gaps in major field crops can help us understand yield variability, yield potential, and the input efficiency of major crops and may indicate appropriate pathways for improving agricultural efficiencies (Fischer et al., 2009; Carberry et al., 2013; Van Ittersum et al., 2013).

Yield gap analysis uses data from field experiments, satellites, simulation models, or a combination of these to understand yield variability and potentiality (Lobell et al., 2005, 2009; Lobell, 2013; Van Ittersum et al., 2013). Recent studies in the US and internationally have shown that yield gaps exists in wheat (*Triticum* spp. L. – Fischer et al., 2009; Carberry et al., 2013; Patrignani et al., 2014), maize (*Zea mays* L. – Egli and Hatfield, 2014a; Rufo et al., 2015), rice (*Oryza sativa* L. – Roel and Plant, 2004; Yang et al., 2008), and soybean [*Glycine max* (L.) Merr. – Egli and Hatfield, 2014b]. Mueller et al. (2012) determined that for most major crops, 60–80% of global yield variability is related to climate, fertilizer application, and irrigation. This variation can be minimized by improving management practices that have the greatest influence on achieving crop yield potential (Dobermann et al., 2003). To date, no information is available to indicate whether the yield and production gaps exist in major field crops in Canada.

The production capacity of farmland is normally calculated as grain yield per unit area, at a standard moisture content. The starting point is actual farm yield (Y_f_), which is average yield achieved in a farmer’s field using the most widely accepted management practices such as sowing date, plant density, nutrient management, and crop protection (Fischer et al., 2009; Van Ittersum et al., 2013). Other measures to quantify these gaps are attainable yield (Y_a_), irrigated yield (Y_i_), and potential yield (Y_p_). Attainable yield is the crop yield grown under optimal management practices (i.e., recommended plant density, non-limiting nutrient condition, effective control of biotic stresses, etc.) in farmers’ fields (Van Ittersum et al., 2013). Y_a_, also referred to as water-limited yield, is the most relevant benchmark for rainfed crops (Evans, 1993; Van Ittersum and Rabbinge, 1997), where crops are limited by water supply and yield is influenced by soil type and field topography. Irrigated yield (Y_i_) represents the yield when a crop is grown under fully irrigated conditions or systems with ample rainfall. In irrigated systems, yield under optimum management is labeled as potential yield (Y_p_) when a crop is grown with nutrient and water non-limiting conditions and effectively controlled biotic stresses (Evans, 1993; Van Ittersum and Rabbinge, 1997). Y_p_ assumes optimal management conditions and that crop growth is determined by genetic characteristics and climatic factors (e.g., solar radiation, temperature, atmospheric CO_2_, light, etc.) (Van Ittersum et al., 2013). The yield gap is primarily the difference between Y_a_ (rainfed crops), or Y_p_ (irrigated crops) and actual farm yields (Y_f_).

Wheat and barley (*Hordeum vulgare* L.) are the principal cereal grains and canola (primarily *Brassica napus* L.) the major oil crop, grown in Canada. Average total national production of spring wheat from 2005 to 2014 was 18.98 million tons (MMt) grown on 6.74 million ha, at an average of 2.82 t ha^-1^, while barley covered 2.95 million ha producing 9.44 MMt, or 3.23 t ha^-1^ (Statistics Canada, 2015). Canola was cultivated on 6.9 million ha with an average productivity of 1.86 t ha^-1^ over the same 10 years span (Statistics Canada, 2015). The proportion of these crops grown in Alberta, Canada are: wheat 34% (2.31 million ha), barley 46% (1.37 million ha), and canola 32% (2.2 million ha) (Statistics Canada, 2015). Although Alberta has the majority of irrigated land in Canada, most of the land is not irrigated.

Wheat, barley, and canola yields vary in Alberta due to genotype (cultivar), location, and management practices (Anbessa et al., 2009; O’Donovan et al., 2011; Harker et al., 2012). Anbessa et al. (2009) reported yields from 5.5 to 8.1 t ha^-1^ for 25 spring barley genotypes grown across six environments in Alberta while O’Donovan et al. (2011) reported yields of 4.1 to 4.4 t ha^-1^ for two malting barleys grown in eight environments. Canola has been a very popular crop among Alberta farmers with the average yield ranging between 2.1 and 4.8 t ha^-1^ (Harker et al., 2012), with the highest yields reported from central Alberta. However, quantification of yield variability of these crops/cultivars at a farmers’ specified level of management, and their potential under optimal management conditions has not been reported. This study was based on the assumption that there exists yield and production gaps in rainfed wheat, barley, and canola in Alberta and that there is a possibility of increasing crop yields by genetic selection and optimal crop management.

The objective of this study was to calculate crop yield and yield potential for the major field crops in Alberta, and to quantify yield gaps. We also identified the gaps that exist between the different measures of yield. Finally, we discuss possible means to narrow the existing gaps. This research offers the opportunity to improve the productivity and the profitability of the three major field crops, wheat, barley, and canola, in western Canada.

## Materials and Methods

### Agroclimatic Conditions in Study Sites

Data on soils and climatic conditions at the research sites were collected from the closest Environment Canada or Alberta Agriculture research station within 5 km of test plots. Soil types and average seasonal precipitation over a period of 10 years are shown in **Table 1**. The five soil types observed in the study locations were Gray Luvisol, Brown Chernozem, Dark Brown Chernozem, Dark Gray Chernozem, and Black Chernozem with the seasonal precipitation received ranging between 197 mm (Acadia Valley) and 288 mm (Lacombe) during the cropping season from May to August.

**Table 1:** Soil types and seasonal precipitation^†^ in the study locations in Alberta.

Location	Geographic coordinates	Soil type		Seasonal precipitation (mm)
			
		Canadian	US	
Beaverlodge	55°20′ N, 119°42′ W	Gray Luvisol	Boralf	227
Greenview	55°06′ N, 117°26′ W	Gray Luvisol	Boralf	236
Smoky River	55°72′ N, 117°20′ W	Gray Luvisol	Boralf	279
Slave Lakes	55°28′ N, 114°77′ W	Gray Luvisol	Boralf	254
Acadia Valley	51°15′ N, 110°20′ W	Brown Chernozem	Aridic Boroll	197
Oyen	51°35′ N, 110°47′ W	Brown Chernozem	Aridic Boroll	206
Hanna	51°63′ N, 111°94′ W	Dark Brown Chernozem	Typic Boroll	225
Castor	52°22′ N, 111°90′ W	Dark Brown Chernozem	Typic Boroll	224
Lethbridge	49°69′ N, 112°83′ W	Dark Brown Chernozem	Typic Boroll	244
Vulcan	50°40′ N, 113°25′ W	Dark Brown Chernozem	Typic Boroll	276
Ft. Kent	54°31′ N, 110°60′ W	Dark Brown Chernozem	Typic Boroll	246
Killam	52°79′ N, 111°85′ W	Black Chernozem	Udic Boroll	248
Irricana	51°31′ N, 113°61′ W	Black Chernozem	Udic Boroll	252
Lacombe	52°46′ N, 113°73′ W	Black Chernozem	Udic Boroll	288
Three Hills	51°70′ N, 113°26′ W	Black Chernozem	Udic Boroll	258
Trochu	51°82′ N, 113°23′ W	Black Chernozem	Udic Boroll	256
Ft. Vermilion	58°39′ N, 116°01′ W	Black Chernozem	Udic Boroll	214
Neapolis	51°65′ N, 113°86′ W	Black Chernozem	Udic Boroll	260
Westlock	54°15′ N, 113°85′ W	Black Chernozem	Udic Boroll	228
St. Paul	53°99′ N, 111°29′ W	Dark Gray Chernozem	Boralfic Boroll	262
Stony Plain	53°53′ N, 114°00′ W	Dark Gray/Black Chernozem	Boralfic Boroll	268


*^†^Ten year’s average of May to August precipitation, data taken from Environment Canada and Alberta Agriculture research stations within 5 km of test plots; Soil descriptions as well as landscape are available at www.agric.gov.ab.ca/asic with the digital maps published at http://agriculture.alberta.ca/acis/maps/agricultural_land_resource_atlas_of_alberta/soil/soil_types_and_classes/soil_groups_big_map.png.*

### Yield and Yield Gap Analysis

The agronomic analysis for this study was based on 18 wheat, 20 barley, and 22 canola genotypes tested at 21 locations across north, south, and central Alberta over a period of 10 years (2005–2014) (**Table 2**). Genotypes were selected for inclusion based on the area planted. The cultivars selected included all those that occupy >1% (10-year’s average) of the total cultivated area (Agriculture Financial Services Corporation [AFSC], 2015).

**Table 2:** Cultivar performance at farmer’s specified level of management under rainfed condition at 21 locations across Alberta over a period of 10 years (2005–2014) (Agriculture Financial Services Corporation [AFSC], 2015).

Wheat				Barley				Canola			

Rank	Cultivar	Yield (t ha^-1^)	CV^†^ (%)	Rank	Cultivar	Yield (t ha^-1^)	CV^†^ (%)	Rank	Cultivar	Yield (t ha^-1^)	CV^†^ (%)
1	AC Foremost (CPS)	4.46	13.9	1	CDC Meredith (M)	4.26	12.5	1	L135	2.38	13.9
2	5700 PR (CPS)	4.02	13.2	2	Vivar (F)	4.10	15.8	2	Invigor 5440	2.22	10.8
3	CDC Stanley (CWRS)	3.70	14.1	3	CDC Austenson (F)	4.05	15.4	3	L130	2.20	9.5
4	AC Crystal (CPS)	3.65	16.7	4	Champion (F)	3.89	11.9	4	L150	2.20	14.3
5	CDC Utmost (CWRS)	3.50	13.9	5	CDC Coalition (F)	3.89	13.6	5	L159	2.20	16.4
6	CDC Go (HRS)	3.42	14.7	6	CDC Thompson (F)	3.78	18.0	6	45H29	2.19	10.5
7	Radiant (HRW)	3.30	11.9	7	CDC Trey (F)	3.62	16.5	7	Invigor 8440	2.14	13.4
8	Stettler (HRS)	3.28	13.0	8	Stander (M)	3.59	16.7	8	VR 9559 G	2.12	10.4
9	Harvest (HRS)	3.24	11.6	9	Newdale (M)	3.57	18.8	9	45S52	2.08	11.5
10	CDC Abound (CWRS)	3.18	12.6	10	Xena (F)	3.52	12.7	10	45H31	2.08	12.6
11	Superb (HRS)	3.07	12.5	11	CDC Copeland (M)	3.49	14.4	11	73–75 RR	2.08	12.8
12	CDC Alsask (HRS)	3.03	17.9	12	Ponoka (F)	3.45	16.2	12	74–44 BL	2.03	12.5
13	AC Inteprid (CWRS)	3.00	16.3	13	CDC Kendall (M)	3.35	15.9	13	Invigor 9590	2.03	13.1
14	CDC Imagine (HRS)	2.96	16.5	14	Sundre (F)	3.35	12.2	14	73–15 RR	1.97	14.8
15	Strongfield (D)	2.82	13.9	15	AC Metcalfe (M)	3.26	14.4	15	L120	1.97	11.1
16	AC Avonlea (D)	2.56	14.8	16	Conlon (F, M)	2.97	16.6	16	VT 500 G	1.96	14.2
17	Lillian (HRS)	2.50	13.4	17	Seebe (F)	2.97	17.7	17	Invigor 5020	1.95	11.8
18	AC Eatonia (HRS)	2.14	13.8	18	CDC Cowboy (F)	2.97	16.8	18	VR 9553 G	1.95	12.6
				19	\vspace*{-9pt}CDC Dolly (F)	2.70	18.1	19	73–45 RR	1.92	13.6
Cultivar Mean (t ha^-1^)		3.20		20	CDC Harrington (F)	2.49	15.5	20	71–45 RR	1.92	13.1
CV (%)		14.2						21	72–65 RR	1.90	14.2
LSD_0.05_		0.48		Cultivar Mean (t ha^-1^)		3.46		22	45H26	1.86	13.8
				CV (%)		15.5					
				LSD_0.05_		0.56		Cultivar Mean (t ha^-1^)		2.06	
								CV (%)		12.8	
								LSD_0.05_		0.26	

*^†^Coefficient of Variation; CPS, Canada Prairie Spring; HRS, Hard Red Spring; HRW, Hard Red White; CWRS, Canada Western Red Spring; D, Durum; F, Feed Type; M, Malt Type.*

Actual farm yield (Y_f_) and irrigated yields (Y_i_) at provincial and regional levels were determined from Agriculture Financial Services Corporation [AFSC] (2015) and Statistics Canada (2015), while the attainable (Y_a_) and maximum attainable (Y_m_) yields of wheat, barley, and canola were derived from the farmers’ managed crop variety performance trials in the same areas (Alberta Regional Variety Trials – Alberta Agriculture and Rural Development [AARD], 2014 and data courtesy of Dean Spaner, Department of Agricultural, Food, and Nutritional Science, University of Alberta) that used optimal crop and nutrient management practices (e.g., soil testing and application of nutrients based on soil types and crop demand, appropriate planting density, effective control of biotic stresses, etc.). Actual, attainable, maximum attainable, and irrigated yields were calculated as described in **Table 3**. The gap between the attainable and actual yields (Y_a_–Y_f_) is a measure of the benefit of proper crop management (i.e., management gap) which would include the proper use of fertilizers and crop protection measures. The gap between the maximum attainable and attainable yields (Y_m_–Y_a_) is a measure of the benefit of using the optimal crop variety (i.e., genetic gap) for that region, while the difference between the maximum attainable and actual yields (Y_m_–Y_f_) is a measure of the benefit of proper crop management plus optimal variety (i.e., total gap), together. Similarly, the gap between the irrigated and actual yields (Y_i_–Y_f_) is a measure of adequate moisture for the crop (i.e., moisture gap), and is often measured by the difference between irrigated and non-irrigated crops. The highest yield (Y_h_) indicates record wheat, barley, and canola yields observed during study period.

**Table 3:** Yield metrics and yield gap calculation in wheat, barley, and canola.

Measures	Definition and limiting factors	Data used	Yield gaps
Actual farm yield (Y_f_)	Average yield of selected cultivars (>1.0%) under rainfed conditions achieved by farmers^†^ **Limiting:** Moisture, Genetics, Crop management, etc.	Regional statistics (Agriculture Financial Services Corporation [AFSC], 2015; Statistics Canada, 2015)	–
Attainable yield or water limited yield (Y_a_)	Average yield for selected (>1.0%) cultivars under rainfed and optimal management conditions, a measure of the benefit of proper crop management^†^ **Limiting**: Moisture, Genetics	On-farm experiments (Alberta Agriculture and Rural Development [AARD], 2014)	*Management Gap*: Y_a_–Y_f_ (Fischer et al., 2009; Van Ittersum et al., 2013)
Max. attainable yield (Y_m_)	Average yield of the top performing cultivar under rainfed and optimal management conditions, a measure of the benefit of genetic selection and optimal crop management^†^ **Limiting**: Moisture	On-farm experiments (Alberta Agriculture and Rural Development [AARD], 2014)	*Genetic Gap*: Y_m_–Y_a_ *Total Gap*: Y_m_–Y_f_ (Lobell et al., 2009)
Irrigated yield (Y_i_)	Average yield for selected (>1.0%) cultivars under irrigated condition, a measure of the benefit of adequate moisture^†^ **Limiting:** Other Factors (e.g., CO${}_{2}$, radiation, temperature)^‡^	Regional Statistics (Agriculture Financial Services Corporation [AFSC], 2015)	Moisture Gap: Y_i_–Y_f_ (Evans, 1993; Van Ittersum and Rabbinge, 1997)
Highest yield (Y_h_)	Highest yield recorded during study period	Anbessa et al., 2009; Harker et al., 2012; Alberta Agriculture and Rural Development [AARD], 2014

*^†^All measurement were made for the same regions within Alberta, over the same 10-years period.*

*^‡^These limiting factors apply to all measurements.*

### Statistical Analyses

An analysis of variance (ANOVA) for grain yield as determined by the genotype, year, and their interactions was done for each location separately using a randomized complete block design. A combined ANOVA was also done from the mean data from each location, to create the means data for the different statistical analyses. The effects of the genotype, location, and year as well as their first and second order interactions were determined from the ANOVA analysis. Genotypes were assumed to be fixed, and year and location effects random. The software package, Agrobase ^TM^ (1990, Agronomix Software Inc.), was used for statistical analyses. Bartlett’s (1947) test was used to determine the homogeneity of variances between environments to determine the validity of the combined ANOVA on the data.

## Results

Actual, attainable, maximum attainable, irrigated, and the highest yield recorded for rainfed wheat, barley, and canola in Alberta are shown in **Table 4**, along with the wheat yield data from Australia, China, and the UK. The 10-years average actual yields (Y_f_) of rainfed wheat, barley, and canola achieved by farmers in Alberta were 3.20, 3.46, and 2.06 t ha^-1^, respectively (Statistics Canada, 2015). Similarly, average attainable yields (Y_a_) were 3.96, 4.32, and 2.68 t ha^-1^, for wheat, barley, and canola, respectively. Therefore, significant management gaps were observed due to difference between actual (Y_f_) and attainable yields (Y_a_) of wheat (an increase of 0.76 t ha^-1^, 24%), barley (0.86 t ha^-1^, i.e., 25%), and canola (0.62 t ha^-1^, i.e., 30%) under rainfed conditions (**Table 4**).

**Table 4:** Actual, attainable, maximum attainable, and irrigated yields of wheat, barley, and canola over a period of 10 years (2005–2014) in Alberta^†^.

Parameters	Alberta			Australia^‡^	China^‡^	UK^#^
				
	Wheat	Barley	Canola	Wheat	Wheat	Wheat
Rainfall^‡^, mm	245	245	245	182	125	287
CV, rainfall, %	32	32	32	48	55	–
Average N rate, kg ha^-1^ (n = 27)	62	57	74	27	260	190
$\upsigma$, N rate, (kg ha^-1^)	27	26	31	28	49	–
Average actual yield, (t ha^-1^)	3.20	3.46	2.06	2.27	6.54	8.2
CV, actual, %	17.4	17.8	13.6	65	12	–
Average attainable, (t ha^-1^)	3.96	4.32	2.68	2.21	8.60	10.4
CV, attainable, %	13.1	14.3	8.8	–	–	–
Average max. attainable yield, (t ha^-1^)	4.68	4.86	2.81	–	–	–
CV, max. attainable, %	15.7	17.5	11.7			–
Average irrigated yield, (t ha^-1^)	4.74	4.57	2.77	2.53	10.30	
CV, irrigated, %	16.2	18.7	17.4	–	–	–
Highest yield recorded, (t ha^-1^)	8.41	10.2^1^	4.80^2^	8.00^3^	10.54^4^	15.6^5^

*^†^calculated using secondary data (Alberta Agriculture and Rural Development [AARD], 2014; Agriculture Financial Services Corporation [AFSC], 2015; Statistics Canada, 2015), ^‡^adopted from Carberry et al., 2013; ^#^Fischer et al., 2009, ^‡^average precipitation from seeding (May) to harvesting (August); ^1^Anbessa et al., 2009; ^2^Harker et al., 2012; ^3^The Commonwealth Scientific and Industrial Research Organization [CSIRO], 2012; ^4^Hou et al., 2012; ^5^Farmers Weekly, 2014.*

The maximum attainable yields of rainfed wheat, barley, and canola were 4.68, 4.86, and 2.81 t ha^-1^, respectively, with the average genetic gaps (i.e., the gap between attainable (Y_a_) and maximum attainable (Y_m_) yields) of 18% (an increase of 0.72 t ha^-1^) in wheat, 12% (an increase of 0.54 t ha^-1^) in barley, and 5% (an increase of 0.13 t ha^-1^) in canola (**Table 4**). Similarly, the total gaps (i.e., the gap between actual (Y_f_) and maximum attainable (Y_m_) yields) were 46, 40, and 36% indicating that combination of optimal management practices and genetic selection can increase grain yields up to 4.68, 4.86, and 2.81 t ha^-1^ for rainfed wheat, barley, and canola, respectively, in Alberta (**Table 4**). The average irrigated yields of wheat, barley, and canola were 4.74, 4.57, and 2.77 t ha^-1^, showing moisture gaps (i.e., the gap between the irrigated yield and the actual farm yield) of 48, 32, and 35%, respectively.

**Table 5** provides an ANOVA for grain yield as determined by the genotype, year, and location as well as their first and second order interactions. Significant variation was observed in the yield of wheat, barley, and canola between genotypes and location under optimal nutrient management. The largest variation was seen between locations (*CV* = 21.3%), probably due to differences in precipitation and soil type (**Table 1**).

**Table 5:** Estimates^†^ of variance components for grain yield, genotypes, and their interactions with location and years under optimal nutrient management.

Components of variance	Wheat			Barley			Canola		
			
	Yield (t ha^-1^)	CV (%)	*F*- Values	Yield (t ha^-1^)	CV (%)	*F*- Values	Yield (t ha^-1^)	CV (%)	*F*- Values
Genotype (σ^2^_g_)	4.86	10.7	3.62^∗^	5.38	11.6	3.55^∗^	3.42	7.5	2.82^∗^
Location (σ^2^_l_)	3.45	21.3	3.12^∗^	3.98	20.7	3.34^∗^	1.92	17.4	1.58^∗^
Year (σ^2^_y_)	3.65	16.4	ns	4.10	18.5	ns	2.05	9.5	ns
Genotype × Year (σ^2^_gy_)	3.94	14.7	3.45^∗^	3.89	15.8	3.18^∗^	2.38	12.6	1.62
Genotype × Location (σ^2^_gl_)	3.51	19.4	3.62^∗^	4.05	20.6	3.22^∗^	2.69	14.2	1.88^∗^
Location × Year (σ^2^_ly_)	3.59	18.4	3.18^∗^	4.00	19.6	3.82^∗^	2.06	15.7	2.12^∗^
G x Y × L (σ^2^_gly_)	3.96	13.1	3.79^∗^	4.32	14.3	3.47^∗^	2.68	8.8	2.82^∗^
Error	24.7			27.3			23.4		


*^†^calculated using data from Alberta Agriculture and Rural Development [AARD], 2014; ^∗^*p* < 0.05; n.s., not significant.*

**Table 6** lists the average actual, attainable, and maximum attainable yields of rainfed wheat, barley, and canola and the percent gap between these components, according to farm location in Alberta. Management gaps (Y_a_–Y_f_) ranged between 12–40, 7–39, and 15–42% whereas the genetic gaps (Y_m_–Y_a_) ranged between 10–32, 11–17, and 4–5% in rainfed wheat, barley, and canola, respectively. The highest yield was observed in areas which receive sufficient rainfall during the cropping season (**Tables 1** and **6**). There was a positive association (*r*^2^ = 0.45) between grain yield and total amount of precipitation over cropping seasons (May to August), showing that location is an another factor in determining grain yield in addition to genotype. Dark Brown to Black Chernozem soils in Lacombe, Stony Plain, Ft. Kent, and Neapolis produced significantly higher yield under optimal management compared to other locations and showed higher gaps in yield (**Table 6**).

**Table 6:** Average actual, attainable, and maximum attainable yields of rainfed wheat, barley, and canola and the percent gap between these components by location in Alberta over a period of 10 years (2005–2014) (Alberta Agriculture and Rural Development [AARD], 2014; Agriculture Financial Services Corporation [AFSC], 2015).

Components	Beaverlodge	Greenview	Smoky River	Slave Lakes	Acadia Valley	Oyen	Hanna	Castor	Lethbridge	Vulcan	Ft. Kent	Killam	Irricana	Lacombe	Three Hills	Trochu	Ft. Vermilion	Neapolis	Westlock	St. Paul	Stony Plain	Average	CV (%)	LSD_0.05_
Wheat
Y_f_ (t ha^-1^)	2.64	2.26	2.72	3.30	2.84	2.98	3.02	2.36	2.96	3.06	4.24	2.98	3.18	4.28	3.25	3.16	2.96	3.46	3.60	3.76	4.19	3.20	19.4	0.48
Y_a_ (t ha^-1^)	3.05	2.62	3.28	4.35	3.56	3.83	3.80	2.74	3.75	3.95	5.35	3.49	4.00	5.22	3.88	3.62	3.82	4.86	4.03	4.56	5.46	3.96	13.1	0.43
Y_m_ (t ha^-1^)	3.50	3.02	4.21	5.57	3.94	4.46	4.67	3.05	4.35	4.43	6.76	3.87	4.42	6.55	4.32	4.35	5.07	5.38	4.80	5.2	6.29	4.68	15.7	0.51
Y_a_–Y_f_ (%)	16	16	21	32	25	29	26	16	27	29	26	17	26	22	19	15	29	40	12	21	30	24	16.5	
Y_m_–Y_a_ (%)	15	15	28	28	10	16	23	11	16	12	26	11	11	25	11	20	32	11	19	14	15	18	12.4	
Y_m_–Y_f_ (%)	32	34	55	69	39	50	55	32	47	45	59	30	39	54	33	38	71	55	34	38	50	46	15.5	
Barley
Y_f_ (t ha^-1^)	2.97	2.95	3.30	3.62	3.54	3.20	3.46	2.68	3.38	3.45	3.95	3.40	3.49	3.91	3.54	3.49	3.45	3.57	3.53	3.75	3.97	3.46	19.2	0.59
Y_a_ (t ha^-1^)	3.60	3.15	3.86	4.95	4.35	4.05	4.12	3.20	4.01	4.35	5.30	3.92	4.06	5.35	4.05	3.90	4.55	4.95	4.70	5.01	5.32	4.32	14.3	0.54
Y_m_ (t ha^-1^)	4.07	3.49	4.33	5.55	4.88	4.57	4.63	3.56	4.50	4.88	5.91	4.35	4.55	6.24	4.55	4.39	5.11	5.55	5.28	5.58	6.08	4.86	17.5	0.62
Y_a_–Y_f_ (%)	21	7	17	37	23	27	19	19	19	26	34	15	16	37	14	12	32	39	33	34	34	25	17.2	
Y_m_–Y_a_ (%)	13	11	12	12	12	13	12	11	12	12	12	11	12	17	12	13	12	12	12	11	14	12	13.4	
Y_m_–Y_f_ (%)	37	18	31	53	38	43	34	33	33	41	50	28	30	60	29	26	48	55	50	49	53	40	16.5	
Canola
Y_f_ (t ha^-1^)	1.92	1.97	2.00	2.13	1.86	1.90	2.00	1.86	1.99	2.12	2.36	2.00	2.12	2.28	2.12	2.07	2.02	2.10	2.05	2.15	2.32	2.06	14.6	0.19
Y_a_ (t ha^-1^)	2.25	2.50	2.51	2.96	2.35	2.42	2.51	2.35	2.32	2.98	3.15	2.5	3.02	3.24	2.42	2.46	2.98	2.80	2.54	2.97	3.15	2.68	8.8	0.17
Y_m_ (t ha^-1^)	2.36	2.62	2.63	3.10	2.46	2.54	2.63	2.46	2.43	3.12	3.30	2.62	3.17	3.40	2.54	2.58	3.12	2.94	2.66	3.11	3.30	2.81	11.7	0.23
Y_a_–Y_f_ (%)	17	27	26	39	26	27	26	26	17	41	33	25	42	42	14	19	48	33	24	38	36	30	12.6	
Y_m_–Y_a_ (%)	6	5	5	5	5	5	5	4	4	4	5	5	5	5	5	5	6	5	4	4	4	5	7.9	
Y_m_–Y_f_ (%)	23	33	32	46	32	34	32	32	22	47	40	31	50	49	20	25	54	40	30	45	42	36	11.4	

*Y_f_, Actual farm yield; Y_a_, Attainable yield; Y_m_, Maximum attainable yield; Y_a_–Y_f_, Management gap; Y_m_–Y_a_, Genetic gap; Y_m_–Y_f_, Total gap.*

Variation also existed among wheat, barley, and canola genotypes selected for yield gaps studies. The rank-wise mean yield of the 18 wheat, 20 barley, and 22 canola genotypes evaluated at 21 sites across Aberta from 2005 to 2014 is given in **Table 2**. The cultivars with the lowest coefficient of variation across the years and locations were, for wheat: Superb, Harvest, Radiant, Stettler, and CDC Abound, for barley: CDC Meredith, Champion, Xena, CDC Coalition, and Sundre, and for canola: L130, Invigor 5440, VR 9559G, and 45H29.

**Table 7** shows the gains in yield that are possible by minimizing yield gaps in wheat, barley, and canola in Alberta alone. Based on this study, the estimated gain in yields of wheat, barley, and canola due to optimal crop management (i.e., management gain) is 1.76, 1.18, and 1.36 million tons, respectively, which was worth $395M, $183M, and $466M (USD) annually, based on 2014–2015 cumulative average crop prices. Production gains which combined genetic selection (i.e., selection of appropriate cultivars) together with an optimal crop management were found to be 3.42, 1.92, and 1.65 million tons of wheat, barley, and canola anually which is equivalent to $769M, $297M, and $564M (USD), respectively (**Table 7**). In other words, the cost of poor genetics (i.e., selection of inappropriate cultivars) was found to be $374M (1.66 million tons) in wheat, $115M (0.74 million tons) in barley, and $98M (0.29 million tons) in canola in Alberta.

**Table 7:** Estimated yield gain in wheat, barley, and canola due to improved crop management and genetic selection under rainfed condition in Alberta.

Yield metrics	Yield (t ha^-1^)	Production (million tons)	Yield Gain (million tons)	Economic values (million US$)^†^
**Wheat (cultivated area = 2.31 million ha)^‡^**				
Actual farm yield (Y_f_)	3.20	7.39	–	–
Attainable yield (Y_a_)	3.96	9.15	1.76^1^	395^1^
Maximum attainable yield (Y_m_)	4.68	10.81	3.42^2^	769^2^
**Barley (cultivated area = 1.37 million ha)^‡^**				
Actual farm yield (Y_f_)	3.46	4.74	–	–
Attainable yield (Y_a_)	4.32	5.92	1.18^1^	183^1^
Maximum attainable yield (Y_m_)	4.86	6.65	1.92^2^	297^2^
**Canola (cultivated area = 2.2 million ha)^‡^**				
Actual farm yield (Y_f_)	2.06	4.53	–	–
Attainable yield (Y_a_)	2.68	5.89	1.36^1^	466^1^
Maximum attainable yield (Y_m_)	2.81	6.18	1.65^2^	564^2^


*^†^2014–2015 cumulative average price of wheat (US$225 per ton), barley (US$155 per ton), and canola (US$342 per ton) (Agriculture and Agri-Food Canada [AAFC], 2015); ^1^management gain = Y_a_–Y_f_; and ^2^total gain = Y_m_–Y_f_; ^‡^Statistics Canada, 2015.*

## Discussion

The analysis of yield gaps is important to identify the potential sources of gains in agricultural yields and to develop solutions to reduce these gaps. These solutions can increase crop yields and optimize the use of applied agricultural inputs. Our studies identified exploitable yield gaps between actual yield (Y_f_), water limited or attainable yield (Y_a_), maximum attainable yield (Y_m_), and irrigated yields (Y_i_) in wheat, barley, and canola in Alberta.

Actual yield varied between locations and years depending on soil type, rainfall, and climate, but was often lower than attainable yield due to poor crop and nutrient management practices. The management gap between Y_f_ and Y_a_ can be narrowed by using site specific crop and nutrient management practices (i.e., soil testing and use of the right amount of fertilizer, planting date, and density, and effective control of biotic stresses) for the choosen cultivars, whereas, the genetic gap between Y_a_ and Y_m_ represents the difference between the top performing variety in that location and the varieties that were chosen. Therefore, the genetic gap can be reduced by selecting the appropriate cultivar, and these gains would continue as new and improved varieties became available. The total gap between Y_f_ and Y_m_ represents the genetic and management gains that can be made using better performing cultivars under optimal management condition and can be minimized when several constraints (e.g., selection of high yielding cultivars, site specific crop and nutrient management practices) are addressed simultaneously. Minimizing this total gap has the potential to offer significant improvements in yield (4.68, 4.86, and 2.81 t ha^-1^ of wheat, barley, and canola, respectively) under rainfed conditions in Alberta.

The moisture gap between Y_f_ and Y_i_ can be narrowed by irrigation for the choosen cultivars. In rainfed system, this gap is mainly determined by factors that are difficult to control including the variation in climatic conditions. The moisture gap, therefore, is usually smaller in seasons with very favorable weather conditions (Pasuquin and Witt, 2007) since adequate moisture allows for the greater utilization of available nutrients, CO_2_, radiation, and temperature, throughout the cropping season.

Yield gaps provide important guidance in the identification of these constraints. If the gaps are large despite using improved management practices, maximum/attainable yields must be limited by an unknown constraint (Pasuquin and Witt, 2007). If the gaps are small, it is usually not economical to aim to fully reducing the gap because of the large amounts of inputs required and the high risk of crop failure and economic losses. A comparative study of yield gaps in wheat in Australia and China suggested that there is only a small gap between actual and attainable yield in Australia while the gap is much wider (32%) in China (Carberry et al., 2013). The small yield gap in Australia, which was determined as reasonable, given the economics and risks, and was attributed to better infrastructure, agricultural institutions, and modernized farms (Carberry et al., 2013). Although the wheat yield gap is still approximately 32% in China, targeted breeding and the availability of higher water and/or N resources have resulted in closer gaps (Carberry et al., 2013) compared to other developing countries.

Yield variation exists among wheat, barley, and canola genotypes and between locations in Alberta (Anbessa et al., 2009; O’Donovan et al., 2011; Harker et al., 2012). Variation between locations is due to crop management, soil types, climate, and availability of moisture during cropping season. The management practices of the actual farm yield in the Alberta Prairies mainly constitutes large-scale production of a few genotypes with effective chemical weed control, higher soil disturbance due to removal of crop biomass after harvest, and reliance on synthetic nutrient formulations which can result in nutrient deficiencies in cropping systems (Martens et al., 2013). Only 20% of the fields in Alberta have been sampled with soil tests, and many of those were only sampled every 3 years (International Plant Nutrition Institute [IPNI], 2010). In general, growers apply fertilizers based on reasons other than available soil N, such as trying to hit yield targets, using past personal experience or what other farmers have applied in their area (Heard, 2011). An unpredictable moisture regime also means than the additional cost of higher inputs may not be rewarded with increased yields.

Yield variation related to soil conditions, fertilizer application, the incorrect genotype, and irrigation can be minimized by adopting site specific optimal crop and nutrient management strategies, which could maximize economic returns. Soil testing, nutrient management planning, and minimum tillage/zero-till have been considered to be the top-performing best management practices (BMPs) in Alberta that can increase expected net revenue by 19, 33, and 35%, respectively, compared to the base model in brown soil zone (Crop Nutrients Council, 2007). Testing soil and manure for nutrient content, the balanced application of manures and mineral fertilizers, adoption of direct seeding, and the application of N fertilizers in bands or placing them safe in seed rows in spring instead of pre-seed broadcasting are several ways to offset costs due to excess applications and may improve yields and nitrogen use efficiency (Alberta Agriculture and Rural Development [AARD], 2015). In recent years, a large percentage of Albertan farmers adopted a minimum or no-tillage system. Producers using minimum tillage, however, identified fewer increases in yields compared to the tillage system, although they typically had improvements in expected net revenue due to reductions in operating costs (Crop Nutrients Council, 2007). Plant population is another factor that limits the crop yield potential of a given environment (Lobell et al., 2009); increased plant population only reduced the yield gap when all other inputs were applied at the supplemental level (Rufo et al., 2015).

Given that high quality agricultural land is being lost to development and producers are moving on to marginal lands for crop production, it is unlikely that more high quality land will be available in the future (Grassini et al., 2013). Furthermore, cereal crop yields, including wheat and barley, have slowed to a growth rate of about 1% annually since the 1990’s, and in some cases, specifically in developed countries, growth of crop yields is close to zero (Fischer et al., 2009). This study has shown that in Alberta, compared to current production levels, gains of 46, 40, and 36% in wheat, barley, and canola, respectively, could be achieved by using more appropriate genetic and management approaches for rainfed farming, leading to a large yield gain and a reduction in production costs.

## Conclusion

This study assessed the yield gaps of the major field crops in Alberta (wheat, barley, and canola) over a period of 10 years (2005–2014), and revealed the possibility of improving yields of the existing cultivars of wheat, barley, and canola by 24, 25, and 30%, respectively, by using proper crop management (i.e., soil testing and use of right amount of fertilizer at right time and place, planting density, and pests and disease management). Average attainable yields from the existing cultivars of wheat, barley, and canola were 3.96, 4.32, and 2.68 t ha^-1^, respectively. Variation was also observed among the genotypes in each location which offers the opportunity of cultivar selection. The combination of optimal crop management practices and selection of location specific cultivars could increase grain yields up to 4.68 t ha^-1^ (46% higher than actual wheat yield), 4.86 t ha^-1^ (40% higher than actual barley yield), and 2.81 t ha^-1^ (36% higher than actual canola yield). This might lead to estimated yield gains of 3.42, 1.92, and 1.65 million tons of wheat, barley, and canola each year worth $769M, $297M, and $564M (USD), respectively, in Alberta.

## Author Contributions

Author TC designed the study, managed the literature review, performed statistical analyses, and produced the first draft of the manuscript. Author AG provided supervision and support in all phases of this research. Both authors have read and approved the final manuscript.

## Conflict of Interest Statement

The authors declare that the research was conducted in the absence of any commercial or financial relationships that could be construed as a potential conflict of interest.
